# Increased Musculoskeletal Surgery Rates During Diagnostic Delay in Psoriatic Arthritis: A Retrospective Cohort Study

**DOI:** 10.3390/diagnostics15091125

**Published:** 2025-04-28

**Authors:** Servet Yolbas, İlyas Gündüz, Mahmut Kara, Emrah Çay, Gülşah Yamancan, Nevra Yalçın, Elif İnanç, Sezgin Zontul, Muhammed Köroğlu

**Affiliations:** 1 Division of Rheumatology, Department of Internal Medicine, Faculty of Medicine, İnönü University, Malatya 44280, Turkey; elif.temelli@hotmail.com; 2 Department of Internal Medicine, Faculty of Medicine, İnönü University, Malatya 44280, Turkey; ilyasgunduz44@gmail.com (İ.G.); mahonicaltes@gmail.com (M.K.); emrah_-cay@hotmail.com (E.Ç.); gulsahaydn@windowslive.com (G.Y.); dr_nevra@hotmail.com (N.Y.); 3 Division of Rheumatology, Department of Physical Therapy and Rehabilitation, Faculty of Medicine, İnönü University, Malatya 44280, Turkey; sezginzontul@hotmail.com; 4 Department of Orthopaedics and Traumatology, Faculty of Medicine, İnönü University, Malatya 44280, Turkey; m.koroglu91@gmail.com

**Keywords:** psoriatic arthritis, musculoskeletal system, orthopaedic procedures, surgery, delayed diagnosis

## Abstract

**Background/Objectives**: Delayed diagnosis in psoriatic arthritis (PsA) is associated with significant health consequences. We hypothesize that musculoskeletal (MSK) surgery rates may be higher during the diagnostic delay period. This study aimed to compare the frequency of MSK surgeries in PsA patients during the period of diagnostic delay with the frequency of MSK surgeries post-diagnosis. **Methods**: This retrospective cohort study included PsA patients who fulfilled CASPAR criteria and were followed up on in our outpatient clinic. The pre-diagnosis symptomatic period was considered as the period of diagnostic delay. Data on MSK surgeries were obtained from patient records. The annual number of surgeries was calculated separately for the diagnostic delay and post-diagnosis periods. **Results**: The study included 84 PsA patients. The mean diagnostic delay in PsA patients was 7.49 years. During this period, 27.4% of patients underwent at least one MSK surgery. The mean annual number of MSK surgeries was significantly higher during the diagnostic delay period compared to the post-diagnosis period (Z = −3.18, *p* = 0.001, r = 0.35). **Conclusions**: Following PsA diagnosis, a reduction in MSK surgery rates was observed compared to during the diagnostic delay period. This suggests that inflammatory symptoms in PsA patients, which could have been managed with medical therapy, may have led to avoidable MSK surgeries. These findings highlight the potential for early diagnosis to reduce the rate of musculoskeletal surgery and associated healthcare costs.

## 1. Introduction

Psoriatic arthritis (PsA) is a chronic inflammatory disease associated with psoriasis (PsO), affecting both peripheral joints and the axial skeleton, often leading to disability. PsO’s prevalence ranges between 0.5% and 5%, with PsA developing in 6–42% of PsO patients [1,2,3].

PsA is characterised by heterogeneous musculoskeletal (MSK) involvement and diverse clinical phenotypes. In 15% of patients, MSK manifestations occur in the absence of PsO [3,4]. Unlike rheumatoid arthritis (RA), PsA is often associated with milder joint pain and may present with normal acute phase reactants in nearly half of patients [4,5].

PsA can present with a variety of radiological features––including inflammatory, erosive and osteoproliferative changes––particularly in mechanically stressed regions. Due to its heterogeneous presentation, PsA is frequently misdiagnosed, most commonly as osteoarthritis or axial skeletal disorders. These diagnostic errors often lead to significant delays in establishing the correct diagnosis and initiating appropriate treatment [6].

With recent advances in PsA pathogenesis, novel pharmacologic treatments have led to improved remission rates [7,8]. However, extant studies have found that healthcare costs begin increasing approximately five years before diagnosis, with MSK surgeries contributing significantly to these costs [9,10,11,12,13]. While previous studies have reported MSK surgery rates ranging between 2% and 48% among PsA patients, most studies focussed on post-diagnosis surgery, with limited data from the pre-diagnosis period [12,13,14,15,16,17,18,19,20].

Given the high frequency of MSK surgeries before PsA diagnoses, we hypothesise that delayed diagnosis may contribute to unnecessary surgical interventions. This study aims to compare the annual frequency of MSK surgeries among PsA patients during the period of diagnostic delay with that of the post-diagnosis period. This study provides insight into the relationship between early diagnosis and MSK operations among PsA patients, as well as the need for preventive studies in clinical practice.

## 2. Materials and Methods

### 2.1. Study Design and Patients

This is a retrospective cohort study that compares the annual frequency of musculoskeletal operations among PsA patients during the symptomatic pre-diagnosis period with the post-diagnosis period. The study sample comprised 84 patients with PsA who met the CASPAR classification criteria and were followed up on at the Rheumatology Clinic at Inonu University, Faculty of Medicine. Patients younger than 18 and older than 65 were excluded. The symptomatic period in PsA is characterised by the presence of one or more of the following symptoms: inflammatory-type lower back or hip pain (e.g., morning stiffness, worsening with rest); peripheral joint pain and/or swelling; dactylitis (diffuse swelling of an entire finger or toe); enthesitis (particularly pain at tendon or ligament insertion sites); and/or coexisting episodes of uveitis. Approval for this study was obtained from the Inonu University Faculty of Medicine Clinical Research Ethics Committee (Ethics Committee No. 2019/133). Informed consent was obtained from the patients participating in this study. All patient data were anonymised before analysis to ensure confidentiality and to protect patient privacy.

### 2.2. Collection of MSK Surgery and Other Clinical Data

Demographic, medical treatment, laboratory and radiological data on the patients were recorded in the patient and follow-up files. Records on all previous MSK operations were obtained from each patient’s clinical history and medical records. The pre-diagnosis symptomatic period was viewed as the period of diagnostic delay. The total number of MSK operations during the pre-diagnosis period was divided by the number of years in that period to calculate the annual surgery rate. Similarly, the number of MSK operations during the post-diagnosis period was divided by the year of diagnosis, and the annual number of operations during the post-diagnosis period was calculated.

### 2.3. Statistical Analysis

Data were analysed using SPSS software (Version 26; IBM Corp., Armonk, NY, USA). Descriptive statistics were used to summarise the data. The variables’ conformity to normal distribution was evaluated using the Kolmogorov–Smirnov test, and homogeneity of variance was evaluated using Levene’s test. Categorical variables were expressed as numerical percentages. Continuous variables with normal distribution were expressed as mean ± standard deviation (SD), while those without normal distribution were reported as medians (minimum–maximum). The McNemar test was used to analyse categorical variables. Furthermore, the Wilcoxon signed-rank test was used to compare ordinal nonparametric data between dependent groups. Statistical significance was set at a *p*-value of 0.05, and the results were supported by confidence intervals.

## 3. Results

### 3.1. Demographic Data/Baseline Characteristics

**Figure 1:**
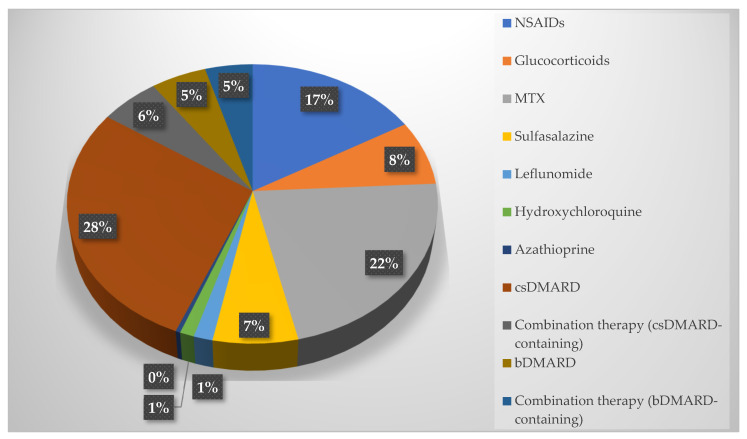
Medical treatments used by PsA patients (compared ratios of drugs among themselves).

The study sample comprised 84 PsA patients, whose demographic and clinical characteristics are summarised in Table 1. The patients’ mean age was 47 ± 11 years, and 88.1% of the patients were female while 11.9% were male. PsA patients had a mean diagnosis duration of 3.39 years and a mean diagnostic delay of 7.49 years. Both axial and peripheral involvement were present in 76.2% of patients. While 41.7% of the patients had PsO themselves, 67.9% had a family history of PsO. Medication usage among the patients revealed that the most frequently utilized conventional synthetic disease-modifying antirheumatic drug (csDMARD) was methotrexate (*n* = 59, 70.2%), followed by sulfasalazine (*n* = 18, 21.4%), leflunomide (*n* = 4, 4.8%), hydroxychloroquine (*n* = 3, 3.6%) and azathioprine (*n* = 1, 1.2%). Biologic DMARDs (bDMARDs) were employed by 16.7% of the patients (*n* = 14), with 14.3% (*n* = 12) receiving combination therapy that included a bDMARD. Additionally, nonsteroidal anti-inflammatory drugs (NSAIDs) were used by 52.4% of the patients (*n* = 44), and glucocorticoids were used by 23.8% (*n* = 20). In addition, the percentages of the drugs used by PsA patients in proportion to each other are given in Figure 1.

### 3.2. MSK Operation Data

A total of 23 patients (27.4%) had at least one MSK operation in the symptomatic period before and after the diagnosis (Table 1). While 39 of the PsA patients underwent MSK surgery in the pre-diagnosis symptomatic period, three of them underwent MSK surgery in the post-diagnosis period. A total 25% of the patients during the pre-diagnosis symptomatic period and 3.6% during the post-diagnosis treatment period had at least 1 MSK operation (Table 2). During this period, one patient underwent five MSK surgeries, while two patients underwent three surgeries each. Only one of the 21 patients who had undergone an MSK operation during the delayed diagnosis period underwent an MSK operation again in the post-diagnosis period (medical treatment). The two patients who underwent surgery after the diagnosis did not have any MSK operation during the delayed diagnosis period. Of those who underwent MSK surgery, 52.4% had operations related to the peripheral MSK system, while 47.6% had spine operations. Of those who underwent peripheral MSK operations, 9% were joint-sacrificing operations (Table 3). Six of the operations related to peripheral CNS structures in PsA patients were performed as soft tissue operations (three tendon operations, one bursitis operation and two carpal tunnel syndrome operations).

The mean number of MSK operations for PsA patients during the pre-diagnosis symptomatic period was significantly higher than during the post-diagnosis period (<0.001, Z = −3.952). Furthermore, the pre-diagnosis symptomatic period was longer than the post-diagnosis period, so statistical tests were performed again after calculating the mean number of MSK operations per time unit (year). Thus, it was possible to compare the number of MSK operations per time unit. In this way, the mean number of MSK operations per year was significantly higher during the symptomatic period before diagnosis than during the treatment period after diagnosis (Z = −3.18, *p* = 0.001, r = 0.35) (Table 2). No significant correlation was found between the number of operations and patient age, PsA symptom duration, PsA diagnosis duration and PsO rash duration among patients who underwent MSK operations during the diagnostic delay period (*p* > 0.05 for all).

Demographic, clinical and treatment characteristics of patients with and without MSK operations were compared (Table 4). MTX intake frequency was significantly higher in the group who underwent MSK operations than in the group without MSK operations (*p* = 0.040). No significant difference was found between the two groups in terms of other data (*p* > 0.05 for all) (Table 4).

### 3.3. Subgroup Analyses 

**Figure 2:**
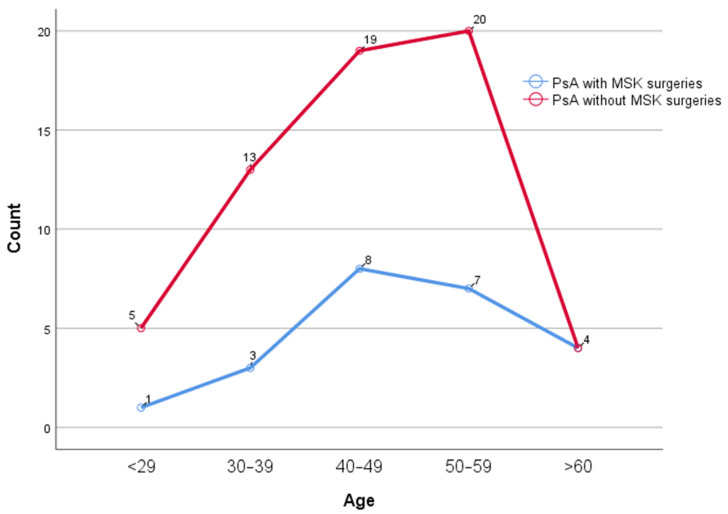
Number of MSK surgeries according to age range.

No significant difference in the number of MSK operations between age decades (*p* = 0.677) (Figure 2) was found. The significant change observed in the presence of MSK surgeries before and after diagnosis is particularly evident in the female patient group (*p* < 0.001, decrease from 25.68% to 4.05%). Among female PsA patients, the number of operations during the delayed diagnosis period was significantly higher than during the post-diagnosis period (*p* < 0.001, Z = −3.748). The number of operations per year during the period of delayed diagnosis was significantly higher among female PsA patients than during the post-diagnosis period (*p* = 0.001, Z = −2.923). However, among male PsA patients, no significant difference was found between the pre-diagnosis symptomatic period and the post-diagnosis period in terms of number of operations and number of operations per year (*p* > 0.005 for both) (Table 5). In patients with axial + peripheral joint involvement, the change in MSK surgery rates before and after diagnosis was statistically significant (*p* = 0.001, 25.00% vs. 3.13%). Although a decrease in post-diagnosis operation rates was also observed in patients with axial and peripheral involvement, this decrease did not reach statistical significance in axial involvement (*p* = 0.250) (Table 5). There was no association between psoriatic rash or nail involvement and MSK surgery (for all, *p* > 0.05). In patients with a family history of PsO, the change in MSK surgery rates before and after diagnosis was quite prominent and statistically significant (*p* < 0.001, decrease from 31.71% to 2.44%). In patients without a family history of PsO, a decrease in operation rates was also observed, but the statistical significance level of this change was lower (*p* = 0.109, decrease from 18.60% to 4.65%) (Table 5). No association was found between bDMARD, csDMARD or their combined use and MSK surgeries (for all, *p* > 0.05).

Generally, a significant decrease in MSK operation rates was observed among all patient groups after diagnosis, and this decrease was found to be statistically significant, particularly among female patients, patients with axial + peripheral joint involvement and patients with a family history of PsO.

## 4. Discussion

In our study, the mean number of MSK surgeries per year was significantly higher during the period of delayed diagnosis than during the period of medical treatment after diagnosis. Furthermore, a 7.49-year delay was found in diagnosis of PsA. This is the first study to compare the mean annual frequency of MSK surgeries among PsA patients during the period of delayed diagnosis with that during the post-diagnosis period.

PsA may affect all joints in the body and follows a variable clinical course [21]. PsA may lead to different pictures developing as a result of intertwined inflammatory, erosive or osteoproliferative changes in the MSK system. Therefore, it overlaps clinically with many degenerative and inflammatory diseases. This leads to the diagnosis of different mimetic diseases other than PsA and, thus, delays diagnosis of PsA for years [4,6,19,22,23,24].

Previously, PsA was viewed as a relatively benign disorder but, in recent years, it has been demonstrated that most PsA patients develop erosive arthritis. Structural damage to the joints of PsA patients has been found to be similar in magnitude and effect to that seen in patients with RA [16]. A delay in diagnosis for patients with PsA leads to progression of the inflammatory process and permanent structural damage to the MSK structure over time [13]. Both peripheral and axial skeletal involvement may occur, so structural and functional disorders may be observed in these areas. Approximately two-thirds of PsA patients with peripheral joint involvement who followed up for five years showed radiographic erosions [13]. Another study found that destructive arthritis occurred in approximately 25% of patients presenting with oligoarticular arthritis and in approximately 65% of patients presenting with polyarticular disease [21]. Haroon et al. found that a six-month delay in diagnosis among PsA patients led to an increased risk of joint erosion and deterioration in long-term physical function outcomes and quality of life [25]. Patients with PsA impose a substantial economic burden on the healthcare system. Extant studies have found that healthcare costs begin to rise approximately five years prior to a diagnosis of PsA and peak at the time of diagnosis [9,10]. The importance of early diagnosis and appropriate adequate treatment to slow the progression of PsA and improve quality of life has been emphasised in many studies [25].

In the years preceding diagnosis of PsA, it is important to identify, prevent or manage both PsA risk factors and PsA-related long-term complications. It has been suggested that there may be a critical period during which we can identify and intervene in patients with early PsA to improve PsA-related complications [9]. Some of the musculoskeletal findings related to PsA may lead to irreversible structural damage, while others may lead to inflammatory changes that can be reversed with medical treatment. With the introduction of new advanced treatment options in the past few decades and demonstration of their effects on radiological progression, this issue has become a focus of interest [13,16,26].

PsA patients have an increased risk of needing MSK surgeries due to progressive MSK system involvement, which varies based on the study methodology and location [13,14,15,16,17,18]. In the Danish registry, the occurrence of joint surgery among PsA patients was found to be twice as high as the general population cohort, and it was demonstrated that this risk increased exponentially as the duration of PsA diagnosis increased. Furthermore, the risk of needing surgery was found to be higher among patients aged 18–40 with diagnosed PsA (22%) than among patients in the general population cohort aged 60 and up (20%) after 15 years of follow-up [16].

A significant proportion of our patients with PsA underwent more MSK surgeries during the delayed diagnostic period compared with the post-diagnostic period. Thus, a significant decrease was found in the number of operations per year during the post-diagnosis and post-treatment periods. As mentioned previously, although many studies have demonstrated that PsA patients are at an increased risk of needing MSK surgeries, our study is the first to compare annual frequency of operations during the pre-diagnosis symptomatic and post-diagnosis periods. Here, calculation of the number of MSK surgeries per unit of time makes the frequency of operations more accurate and comparable. In previous studies, the number of operations across varying durations made objective evaluation very difficult.

In a study by Haque et al., frequency of orthopaedic interventions among PsA patients before and after diagnosis was evaluated. In that study, 63.92% of all operations were performed after diagnosis, and 36.07% were performed before diagnosis of PsA. However, the duration of these symptoms before diagnosis was not specified in that study. Furthermore, these operations’ duration and frequency for a certain unit of time were not calculated [18]. In our study, frequency of MSK surgeries before and after diagnosis was determined and compared per one-year time unit. This provides the advantage of being more objective and comparable.

MSK surgeries on PsA patients are usually performed to relieve pain caused by inflammation or structural defects and to restore physical function [13,16]. In different studies, cumulative disease activity, number of tender/swollen areas and high erythrocyte sedimentation rate (ESR), which are indicators of increased inflammation, have been associated with increased MSK surgical risk [13,17,18]. A study by Kwok et al. found that a significant proportion of surgical indications was due to inflammatory variables (42%). High ESR and active inflamed joint numbers (high disease activity markers), which are indicators of uncontrolled inflammation in PsA, were found to be associated with MSK surgery frequency and the risk of undergoing major operations [13]. In the same study, 11% of the patients were subjected to revision surgery after the first operation, which was associated with continuation of the inflammatory process [13]. Another study found that operations were usually performed in the most severe disease state [17]. Patients with PsA have a higher overall risk of undergoing total hip arthroplasty and total knee arthroplasty than the general population, but temporal trends suggest that the risk is decreasing for patients diagnosed in recent years. It has been emphasised that collaboration between rheumatologists and orthopaedic surgeons should be strengthened because interdisciplinary evaluation is very important to improving the outcomes of PsA patients undergoing orthopaedic surgery [14].

Extant studies have found an increase in MSK surgery frequency with disease year. In our study, lower annual MSK surgery frequencies were found after PsA diagnosis. Furthermore, more operations were performed during the early symptomatic period before diagnosis, in our study, which indicates that some of these operations were related to inflammation and that suppression of inflammation and symptoms with medical treatment reduced the number of operations. This suggests that early diagnosis may prevent MSK surgeries associated with these reversible inflammatory conditions. A multidisciplinary approach may make a very important contribution here. In particular, units that perform MSK surgeries, such as orthopaedics and neurosurgery, which evaluate patients with early diagnosis questionnaires for PsA risk before the operation and refer risky patients to rheumatology clinics, may contribute to early diagnosis of PsA and prevent MSK surgeries with treatment [27]. Interestingly, one study found a protective effect from the Psoriasis Area Severity Index on operations among PsA patients. In our study, no correlation was found between the presence of a psoriatic rash and nail involvement, and MSK operation status. However, we did not have PASI scores on patients during MSK surgeries, so no evaluation could be made on these scores [13].

In some studies, biological treatments’ effect on MSK operations was not found to be statistically significant. It has been stated that this may be related to the gradual transition to these drugs, which increases the risk of structural damage in diagnosed patients and of osteoarthritis accompanying PsA [13,17]. In our study, no significant relationship was found between medication use and MSK surgeries. This supports the idea that treatment, independent of drug type, affects frequency of operations. Although no significant relationship was found between biological drug use and MSK surgeries in our study, this aligns with previous findings suggesting that delayed initiation of biologics may limit their protective effects against structural damage. The potential benefits of early treatment initiation, particularly with biologics, in preventing surgical outcomes remain an area requiring further prospective evaluation. Interestingly, a higher proportion of MTX use was observed in the surgery group. This may reflect a tendency to initiate MTX in patients with more severe disease presentation, rather than its direct association with surgical risk. Therefore, MTX use in this context might be more indicative of baseline disease severity than treatment inefficacy.

Our study also has some limitations. First, it was conducted in a single medical centre, thereby limiting our findings’ generalizability to other populations. We acknowledge the limitation regarding our study’s single-centre design and the exclusion of individuals outside the 18–65 age range. These factors may affect our findings’ generalizability to broader populations. Specifically, the results may not fully represent younger or older individuals with PsA, nor patients from different geographical or healthcare contexts. Furthermore, centre-specific factors, such as local referral patterns and surgical practices, may have influenced the observed rates of MSK surgeries, further limiting our results’ applicability to other settings. Second, the cross-sectional design prevents this study from evaluating any of the clinical outcomes in the long term. Third, our patient numbers were too small to assess different disease patterns and treatment subgroups of PsA. Fourth, the use of patient records for MSK surgical history may have introduced recall or documentation bias. Furthermore, no detailed assessment was made of MSK surgery types and aetiology. Additionally, missing data were handled using complete-case analysis, and variables with significant levels of missingness were excluded from inferential analyses. In addition, no detailed assessment was made of the types and aetiology of MSK surgeries. The fact that the majority of our patients were women limited the evaluation of the gender differences in this outcome. Future multi-centre studies with a more diverse age range could provide a clearer understanding of how diagnostic delays and MSK surgery rates may vary across different populations. Studies evaluating the annual frequency of MSK surgeries may more objectively and comparably reveal the risks of such procedures and their association with different treatment options.

## 5. Conclusions

In conclusion, our study demonstrates that delayed diagnosis of PsA is associated with a significantly higher rate of MSK surgeries. While an increase in MSK surgeries may be expected over the course of PsA due to progressive joint damage, our findings indicate that timely diagnosis and medical treatment significantly reduce these surgical interventions’ frequency. This suggests that many of the surgeries performed during the pre-diagnosis symptomatic period might have been avoidable with earlier recognition and treatment. Evaluating patients scheduled for MSK surgeries—particularly in orthopaedics and neurosurgery—for possible PsA may help identify cases earlier and prevent potentially reversible inflammation-related procedures. These results emphasise early PsA detection’s critical importance in mitigating surgical risk and reducing healthcare resource utilisation. Future prospective studies should explore whether early initiation of advanced therapies, particularly biologics, can prevent the need for MSK surgeries in this patient population further.

## Figures and Tables

**Table 1:** Demographic and clinical characteristics of psoriatic arthritis patients.

Variable	Value
Age (years), mean ± SD	47 ± 11
Female/male, *N* (%)	74 (88.1)/10 (11.9)
Duration of symptoms before diagnosis (years), mean ± SD (max–min)	7.49 ± 8.12 (1–50)
PsA duration (years), mean ± SD	3.39 ± 4.26
Involvement site (axial/peripheral/axial + peripheral), *N* (%)	11 (13.1)/9 (10.7)/64 (76.2)
Psoriasis duration (years), mean ± SD	8.31 ± 8.33
Psoriasis (skin involvement), *N* (%)	35 (41.7)
Psoriasis (nail involvement), *N* (%)	28 (33.3)
Family history of psoriasis, *N* (%)	41 (48.8)
Patients with at least one MSK surgery, *N* (%)	23 (27.4)

**Table 2:** Musculoskeletal (MSK) surgeries during the symptomatic period before diagnosis and the post-diagnosis treatment period.

	Pre-Diagnostic Symptomatic Period	Post-Diagnosis Period	*p*-Value ^† ^
Number of patients with at least one MSK surgery, *N* (%)	21 (25%)	3 (3.6%)	0.583 ^a^
Patients with one MSK surgery, *N* (%)	8 (9.5%)	3 (3.6%)	-
Patients with ≥2 MSK surgeries, *N* (%)	13 (15.5%)	0 (0%)	-
MSK surgeries per patient, median (range)	0 (0–5)	0 (0–1)	<0.001 ^b^ (Z = −3.952)
Annual MSK surgeries per patient, median (range)	0 (0–1.67)	0 (0–1)	0.001 ^b^ (Z = −3.182)

^† ^ Kolmogorov–Smirnov test indicated non-normal distribution (*p* < 0.05). Non-parametric methods were used accordingly. ^a^ McNemar test (for paired categorical data), ^b^ Wilcoxon signed-rank test (non-parametric).

**Table 3:** Distribution of surgical sites and type of surgery.

Surgical Sites	*n*	%
Peripheral (total) *	22	52.4
• Knee **	15	35.7
• Ankle	2	4.8
• Hip	2	4.8
• Wrist	2	4.8
• Elbow	1	2.4
Spine (total)	20	47.6
• Lumbar disc operations	12	28.6
• Other spine procedures (vertebral fracture, spondylolisthesis, vertebroplasty, stabilisation, etc.)	8	19.0
Total	42	100.0

* In PsA patients, 6 of the peripheral MSK structure-related surgeries were performed as soft tissue surgeries (3 tendon surgeries, 1 bursitis surgery and 2 carpal tunnel syndrome surgeries). ** Of the knee procedures, 2 were total knee replacements and the others were non-joint-sacrificing surgeries.

**Table 4:** Demographic and clinical characteristics of psoriatic arthritis patients with and without musculoskeletal operation.

Variable	PsA with MSK Surgeries	PsA Without MSK Surgeries	*p*-Values
Age (years), Mean ± SD	49.4 ± 10.9	46.2 ± 11.6	0.257
Female/Male, *N* (%)	21 (91.3)/2 (8.7)	53 (86.9)/8 (313.1)	0.577
Axial involvement, *N* (%)	3 (13)	8 (13.1)	0.916
Peripheral involvement, *N* (%)	3 (13)	6 (9.8)	
Axial and peripheral involvement, *N* (%)	17 (73.9)	47 (77)	
Duration of symptoms before diagnosis (years), Mean ± SD (max–min)	8.2 ± 8.4	7 ± 6.7	0.516
PsA duration (years), Mean ± SD	2.8 ± 2.1	3.6 ± 4.8	0.493
Psoriasis duration (years), Mean ± SD	10.1 ± 8.9	7.6 ± 8.1	0.431
Psoriasis (skin involvement), *N* (%)	10 (43.5)	25 (41)	0.836
Psoriasis (nail involvement), *N* (%)	10 (43.5)	18 (29.5)	0.226
Family history of psoriasis, *N* (%)	13 (56.5)	28 (45.9)	0.611
Medications Used			
NSAIDs, *N* (%)	15 (65.2)	29 (47.5)	0.148
Glucocorticoids, *N* (%)	6 (26.1)	14 (23)	0.763
Methotrexate, *N* (%)	20 (87)	39 (63.9)	0.040
Sulfasalazine, *N* (%)	2 (8.7)	16 (26.2)	0.134
Leflunomide, *N* (%)	0 (0)	1 (6.6)	-
Hydroxychloroquine, *N* (%)	1 (4.3)	2 (3.3)	-
Azathioprine, *N* (%)	0 (0)	1 (1.6)	-
csDMARD, *N* (%)	21 (91.3)	54 (88.5)	0.713
Combination therapy (csDMARD-containing), *N* (%)	6 (26.1)	9 (14.8)	0.227
Biologic DMARD, *N* (%)	1 (4.3)	13 (21.3)	0.063
Combination therapy (bDMARD-containing), *N* (%)	2 (8.7)	10 (16.4)	0.369

**Table 5:** Variation of the presence of MSK surgery in the pre-diagnosis symptomatic period and post-diagnosis period according to gender, type of MSK involvement and presence of psoriasis in the family.

Variables			Gender				Sites of MSK Involvement						Family History of Psoriasis			
			Female	*p* *	Male	*p* *	Axial	*p* *	Peripheral	*p* *	Axial + Peripheral	*p* *	Yes	*p* *	No	*p* *
Presence of MSK surgery	**Pre- diagnosis**	**Yes**	19 (25.68)	**<0.001**	2 (20.00)	0.5	3 (27.27)	0.250	2 (22.22)		16 (25.00)	0.001	8 (18.60)	0.109	13 (31.71)	<0.001
		**No**	55 (74.32)		8 (80.00)		8 (72.73)		7 (77.78)		48 (75.00)		35 (81.40)		28 (68.29)	
	**Post- diagnosis**	**Yes**	3 (4.05)		0 (0.00)		0 (0.00)		1 (11.11)		2 (3.13)		2 (4.65)		1 (2.44)	
		**No**	71 (95.95)		10 (100.00)		11 (100.00)		8 (88.89)		62 (96.88)		41 (95.35)		40 (97.56)	

*: McNemar test.

## Data Availability

The data presented in this study are available on request from the corresponding author.

## Abbreviations

bDMARD	Biologic disease-modifying antirheumatic drug
csDMARD	Conventional synthetic disease-modifying antirheumatic drug
ESR	Erythrocyte sedimentation rate
MSK	Musculoskeletal system
MTX	Methotrexate
NSAID	Nonsteroidal anti-inflammatory drug
PsA	Psoriatic arthritis
PsO	Psoriasis
RA	Rheumatoid arthritis
SpA	Spondyloarthritis
